# Motivations and future plans of the final year students in a Chinese dental school

**DOI:** 10.1186/s12909-022-03156-6

**Published:** 2022-02-09

**Authors:** Chao Xu, Liang Gao, Shuai Zhang, Jiamin Zhang, Chen Li, Dongmei Zhang, Yaping Pan, Jingbo Liu

**Affiliations:** Department of Periodontics, School of Stomatology, China Medical University, Shenyang City, Liaoning province China

**Keywords:** Medical education research, Undergraduate, Planning, Decision-making, Dentistry

## Abstract

**Background:**

Understanding dental students’ future career choice and motivation could provide beneficial references for both educators and students, but there were few studies on students in a Chinese dental school. The study aimed to investigate Chinese final year dental students’ the short-term and long-term plans, motivations, and identify the influence of gender on the future plans.

**Methods:**

A total of 265 final year dental school students of the School of Stomatology, China Medical University from 2016 through 2020 were invited to complete an anonymous, 27-item questionnaire. Moreover, almost all of questions were in multiple-choice formats. Data were categorized and analysed using chi-square comparative analyses.

**Results:**

88.3% of respondents decided to pursue a graduate degree after graduating from dental school. Moreover, the single most important reason influencing their plans was “eligible for better jobs” (42.8%). More females than males studied dentistry (222 vs 111), and gender had an influence on the choice of specialty.

**Conclusions:**

This study listed the selection tendency and influencing factors of students in a Chinese dental school for the reference of educators and students. And the results could raise some useful influence and feedback effect on current health and education policy, and on the career development of practicing dentists or dental students.

**Supplementary Information:**

The online version contains supplementary material available at 10.1186/s12909-022-03156-6.

## Background

Modern dental education in China began in the early twentieth century. Currently, there were about 101 dental schools in China, mainly offering five-year dental bachelor’s degree programs [1]. The 5-year dental program consisted of three stages in chronological order: basic medical science, general medical science, and dentistry stage. The final (fifth) year of dental education program is focused on clinical practice training, which should follow the standards of Clinical Practice China, formulated by the Society of Dental Education of Chinese Stomatological Association [2]. On the base of the views of educational experts in various fields, the students’ feedback, and the experience of the dental curriculum of other countries, the dental educate programs were constantly adjusted in recent years to promote the career change of dentists and oral medical model in China [3–5]. The adjustments of dental education included the promotion of early clinical contact [3], the emphasis on the Standardized Residency Training (SRT) program [4] and the choice of clinical training patterns [5]. These adjustments and the development of the economy had a great impact on students in Chinese dental schools. Through these adjustments, dentists in China improved their overall understanding of oral diseases and comprehensive analysis capabilities, as well as their clinical competence [4]. Besides, with the development of economy, the country has paid more and more attention to oral health and increased its investment in the oral industry, which gave dentists in China a bright future [6]. In China, students in dental schools had a variety of career choices, which included general dentist, specialist dentist, teacher, civil servant, self-employed, etc. [7]. There were two ways for 5-year students to become specialists: one was to participate in residents training programs after graduation, and the other was to participate in the clinical master program, which not only obtained master’s degree but also residents training program certification after qualifying training. Besides, orthodontics and prosthodontics were the most popular specialties, while oral pathology was the most difficult major to recruit students [7].

Many studies have reported the plans of dental school students after graduation. Puryer et al. [8] found that a large proportion (71%) of UK undergraduates expressed a desire to specialist following graduation, with orthodontics being the most popular intended specialty. A study [9] investigated the views of USA final (fourth) year dental school students and found that 58.9% of respondents planned to immediately enter private practice after dental school, and the female were more likely to enter private practice than male (69.0% vs. 51.8%, *p* < 0.05). A subsequent study [10] showed that most American senior dental school students planned to immediately enter private practice (50.5%) or advanced dental education (33.8%). Study carried out in the USA [11] showed that orthodontics was the most popular specialty choice. Yan et al. [12] found that most 8-year stomatology medical doctor program students in China planned to work as clinical dentists of university hospitals in first-tier cities. The 8-year programme was only organized in several well-known dental schools in China, while the 5-year bachelor’s degree dental students made up the majority of dental students. However, there were rare studies on plans of 5-year dental school students after graduation in China.

The most crucial decision for a dental student after graduation was undoubtedly the plan of the future. It was conditioned by many factors such as educational debt, gender, flexible working hours, and, the influence of a family dentist influenced students’ career plans [9, 13]. A study [14] showed a positive image of dental profession was the main factor for students’ pursuing dentistry. In the United Arab Emirates, a study [15] found altruism was the main motivation for a career in dentistry. In Brazil, choice of career and career plans were influenced by factors related to the students’ characteristics and their conception of the profession [16]. On the one hand, investigating students’ future and motivation after graduation would help the educators to know how the educate programs are evolving and whether education and practices are satisfying students’ needs. The educators could then help better select and train dentists by adjusting the education curricula to improve teaching methods and contents. On the other hand, through a detailed study of student’ future plan and motivation, students might come up with a more perfect plan for the future, and effective measures could be taken in the labour market to attract graduates. In other words, it could promote the relationship between graduates and the labour market by studying students’ future plans and motivations.

The medical profession is currently facing one of relevant changes called feminization of medicine, with a growing proportion of female doctors [17]. And the past 25 years have seen an impressive feminization of dentistry in most of the industrialized countries of the world, particularly in Some European countries, such as Germany, Finland and Turkey, where more than 50% of the active dental professionals were women [18]. This trend could be viewed as a success for female emancipation after a long history of limited access for women to higher education and work. However, the feminization of medicine was also considered to be one of the factors leading to the shortage of labour in specific fields such as medical research and surgery, which were less attractive to women [19]. Besides, many studies found the huge difference was exited between male and female students in motivational factors for choosing dentistry. A study [20] showed a statistically significant difference was detected between male and female students (*p* < 0.05) in the desire for helping others and the balance between work and family life. Therefore, with the majority of the current population of dental students being female, investigating dental student’s future plan can identify the possible influence of feminization and can understand the relationship between gender and the future plan.

The career plan was a critical decision for students as it impacted on their future life and it was influenced by several factors. However, little was known about the China undergraduate dental students’ postgraduate plans, as well as the factors that influence their decision. The study aimed to 1) explore the short-term and long-term plans, 2) identify influencing factors of future plans, and 3) investigate whether there is a relationship between gender and the future plans for the final year students at stomatology school, China Medical University from 2016 to 2020.

## Methods

The study was approved by the Institutional Review Board of the School of Stomatology, China Medical University. The final (fifth) year dental students of the School of Stomatology, China Medical University were at the end of their course perceive and exposed to multi-disciplinary clinical practice. CMU had 90 final year dental students of CMU in 2016, 57 in 2017, 60 in 2018, 63 in 2019, as well as 63 final year dental students in 2020. Before the start of the investigation, the invitation letter included the purpose of the survey, the statement of confidentiality, and notice of approval from the Institutional Review Board of CMU, all students were informed orally and by post. Participation was voluntary and anonymous. To obtain maximum participation and minimum disruption of the students, the questionnaire was issued and collected by face to face following the fifth year (final) examinations in June 2016–2020. Of a total of 333 students, 68 were reluctant to respond to the questionnaire, thus they were excluded from the study. Finally, 265 students were enrolled in this study. The written informed consents were obtained from all participants before they filled in the questionnaires.

A questionnaire was designed to assess information about the perceived motivation for career choice, the short- and long-term future plans, and to identify the factors influencing these three aspects of final year dental students. The questionnaire was divided into the following four sections: demographics (Items 1–7), short-term future career choices (Items 8 and 9), and long-term future plans (Items 18–27). In additional, the respondents who stated “pursue a graduate degree” as their short-term future plan, were invited to fill in the items 10 to 17, including their specialty choice, the factors influencing specialty choice, time of decision, and master degree type choice. When asked their plans and first choice after graduation, if the responders chose the other option rather than “pursue a graduate degree”, they could skip the item 10 to 17, and answer item 18 directly. The questionnaire included 24 multiple-choice questions and three descriptive questions, and the average time needed to fill out the questionnaire was 10 min.

The data were processed using Statistical Package for the Social Sciences (SPSS), version 23.0 for Windows (SPSS Inc., Chicago, IL, USA). Descriptive analysis was undertaken to present an overview of the findings from this survey. Statistical significance was calculated by using chi-square comparative analyses. A *p*-value less than 0.05was considered statistically significant.

## Results

### Demographic information

A total of 265 of the 333 final year dental school students completed the survey, for a response rate of 79.6%. The surveys were carried out between June 2016 and June 2020, and all students were surveyed in final year. All respondents had entered dental school immediately after high school, and were between the ages of 19 to 26 years, and single. The respondents’ sex, family income, and the source of tuition and living expenses were given in Table 1. Of the respondents, 178 (67.2%) were female, and 78 (29.4%) were male. When asked the item regarding family income, the majority of these respondents were mostly distributed in between 50,000–100,000 RMB (27.2%) and between 12,000–50,000 RMB (37.7%) (1 RMB = 0.157 Dollars). When asked about the source of their tuition and living expenses, most respondents reported that they depended on parent and family’s financial support (83.0%).

**Table 1:** Demographic information of the respondents

Survey Item	Number (%)
Sex	
Male	78 (29.4)
Female	178 (67.2)
Deletion	9 (3.4)
Family income (RMB)	
Less than 12,000	40 (15.1)
12,000–50,000	100 (37.7)
50,000–100,000	72 (27.2)
100,000–500, 000	47 (17.7)
More than 500,000RMB	4 (1.5)
Deletion	2 (0.8)
The source of tuition and living expenses	
Parents and family	220 (83)
Loan	17 (6.4)
Part-time job	4 (1.5)
Scholarship	0 (0)
Other	24 (9.1)

^a^(1 RMB = 0.157 Dollars)

Chi-square analysis showed that there were gender differences in the choice of postgraduate majors (*p* < 0.05), which were described in detail in the results of the last part. But beyond this, no difference between the sexes (*p* > 0.05) for other categories of results was detected. No significant difference between family income categories, between home location categories, and between the source of tuition and living expenses (*p* > 0.05) for any categories of results via Chi-square analysis.

### Short-term future plan

**Fig. 1:**
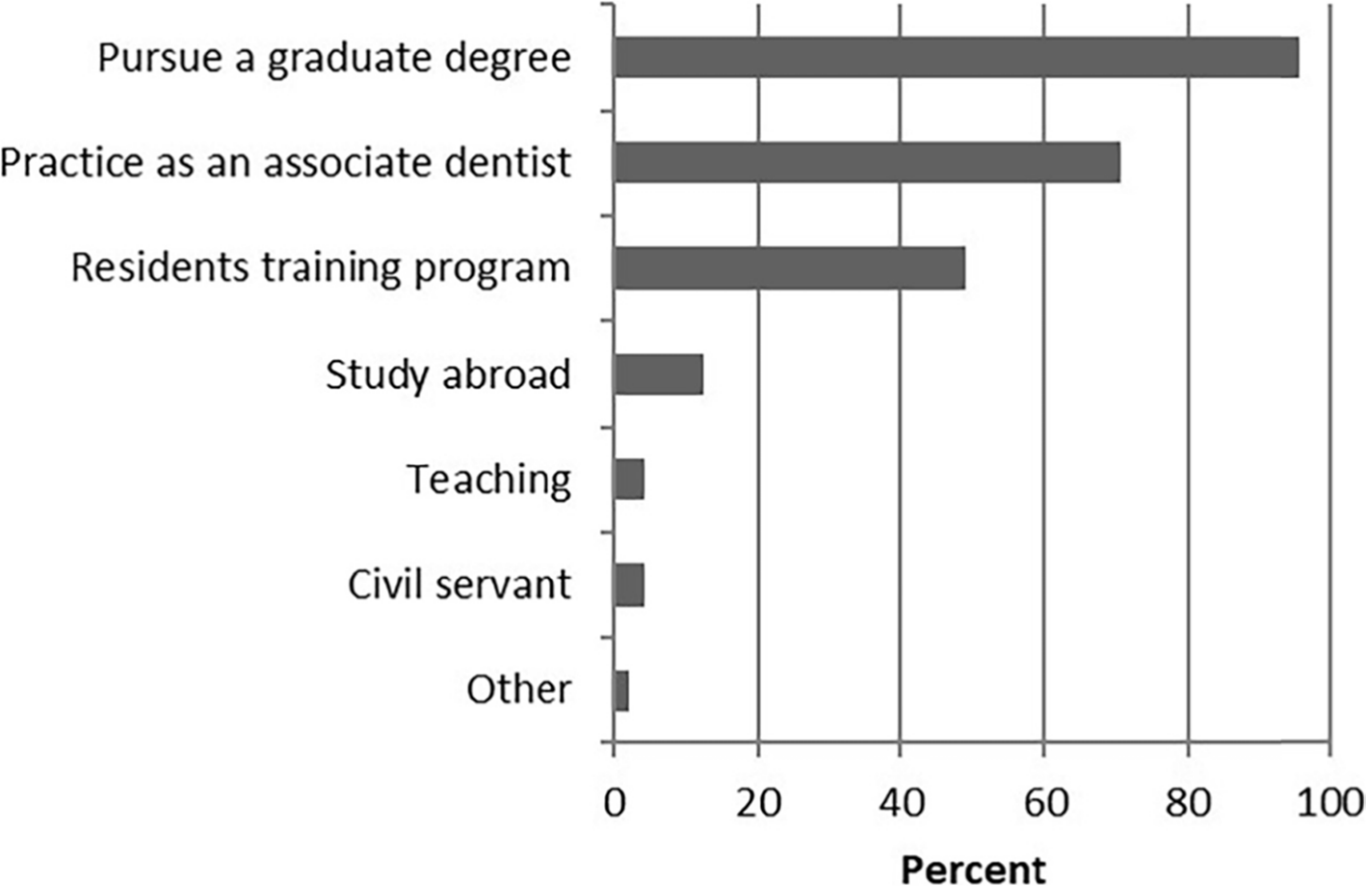
Responses to the question: “What were your plans after graduate”. Respondents could select all answers that applied

Responses to the item regarding the short-term future plan after graduation were shown in Fig. 1. “Pursue a graduate degree” (95.8%) and “practice an associate dentist” (70.6%) were the most popular choice for dental students’ future plans after graduation, followed by “apply for residency training program” (49.1%). When asked about their first choice of future plans after graduation, again the greatest percentage of total respondents stated that they intended to pursue a graduate degree (88.3%). 8.7% reported that they intended to practice as an associate dentist, and only 1.5% planned to study abroad.

### ***Factors influenced*** pursue a graduate degree after graduation

**Fig. 2:**
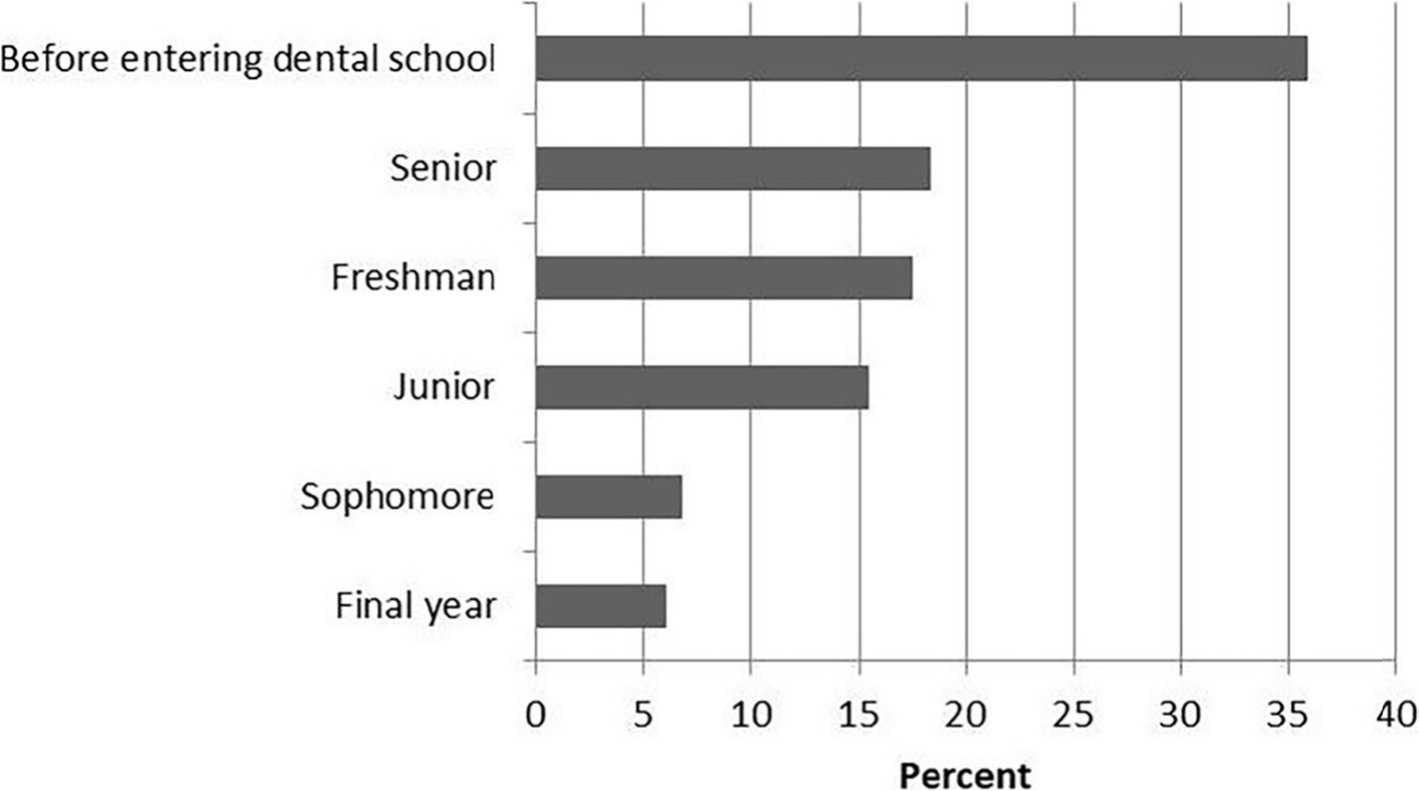
Responses to the question: “when did you decide to pursue a graduate degree”

The vast majority of those respondents decided to pursue a graduate degree after graduating from dental school. Therefore, a total of 254 respondents who chose “pursue a graduate degree” as their future plan and/or the first choice after graduation, were invited to fill in the 7 additional items including the factors influencing “pursue a graduate degree”, their specialty choice of graduate program, the factors influencing specialty choice, time of decision, and graduate degree type choice. As shown in Fig. 2, the decision to pursue a graduate degree was made by 35.9% of respondents before entering dental school. 18.3% had decided while they were senior, 17.5% had decided while they were freshman, and following by decision were made while they were junior (15.5%), sophomore (6.8%), and final year (6.0%).

**Fig. 3:**
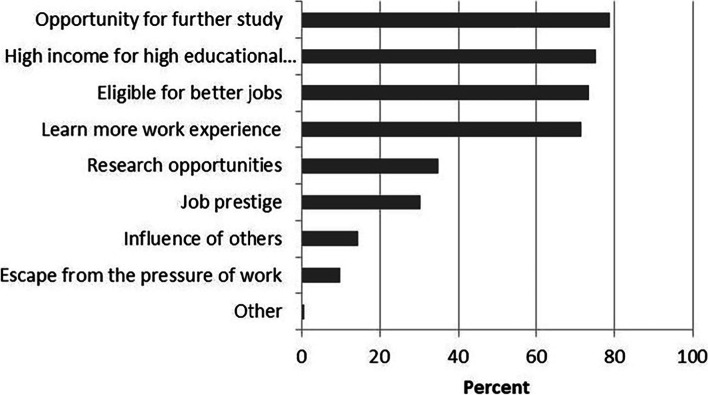
Responses to the question: “what were the main reasons influencing your decision to pursue a graduate degree”. Respondents could select all answers that applied

The main factors influencing the respondent’s decision to pursue a graduate degree were “opportunity for further study” (78.8%), “high income for high educational background” (75.2%), “eligible for better jobs” (73.6%) (Fig. 3). However, when asked about their single most important factor influencing their decision to pursue a graduate degree, the greatest percentage of respondents chose “eligible for better jobs” (42.8%). After this was “opportunity for further study” (21.1%).

**Fig. 4:**
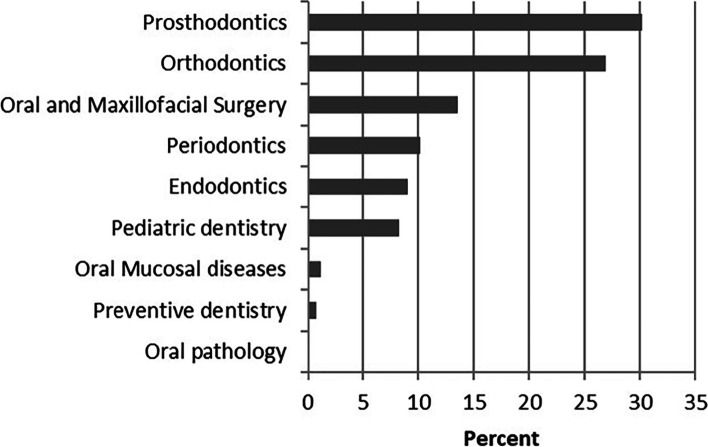
Response to the question: “what was first specialty choice of graduate program”

Figure 4 showed the respondents’ specialty choice for the graduate program. Among the respondents who decided to pursue a graduate degree, only four respondents chose the oral science master program. Among the students who chose a clinical master program, 30.2% chose prosthodontics as the specialty choice of graduate program. Orthodontics (26.9%) and oral and maxillofacial surgery (13.5%) were also identified as the popular specialty choice of graduate program. After these were periodontics (10.2%), endodontics (9.0%) and paediatric dentistry (8.2%). And only 0.8% chose preventive dentistry as the specialty choice of graduate program.

**Fig. 5:**
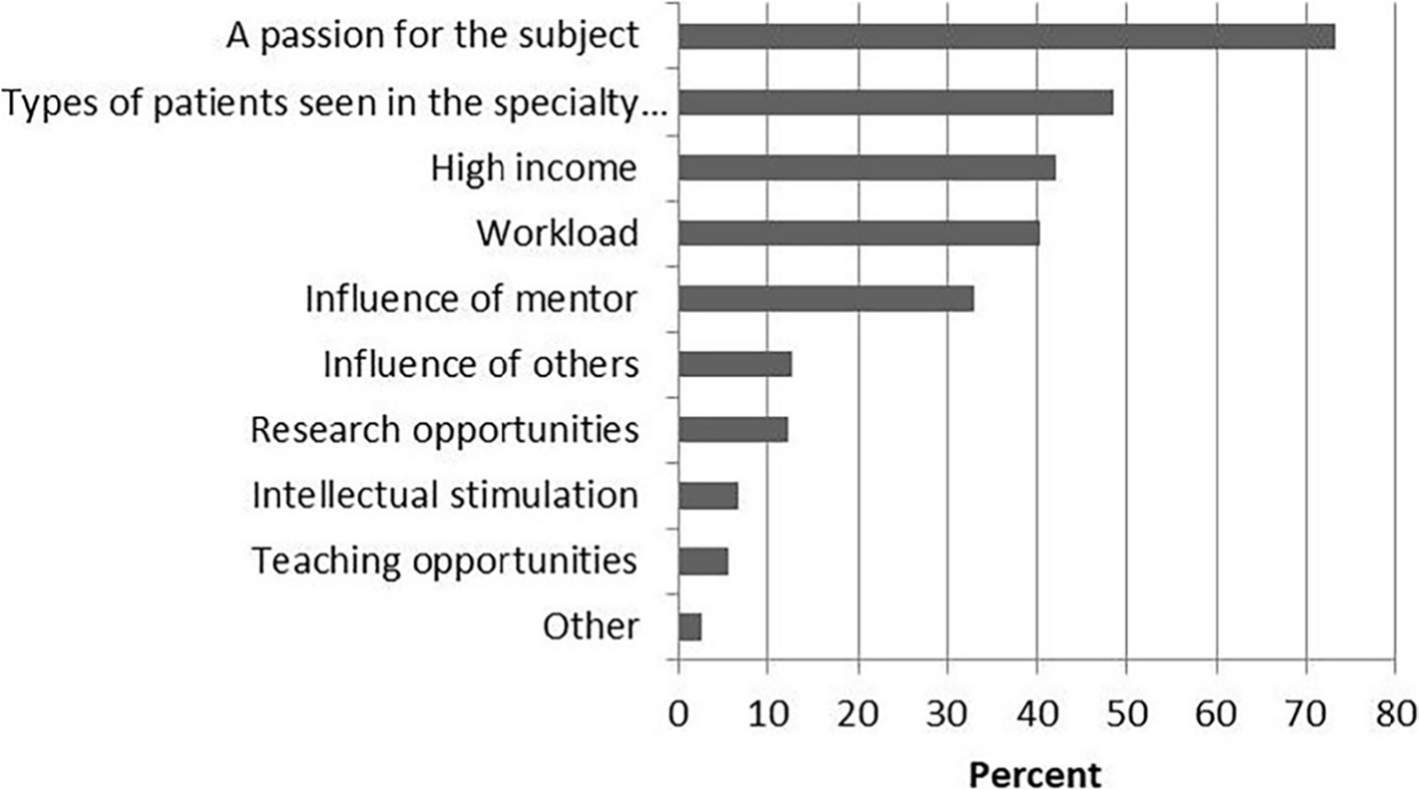
Responses to the question: “what were main factors influencing your specialty choice of graduate degree”. Respondents could select all answers that applied

**Fig. 6:**
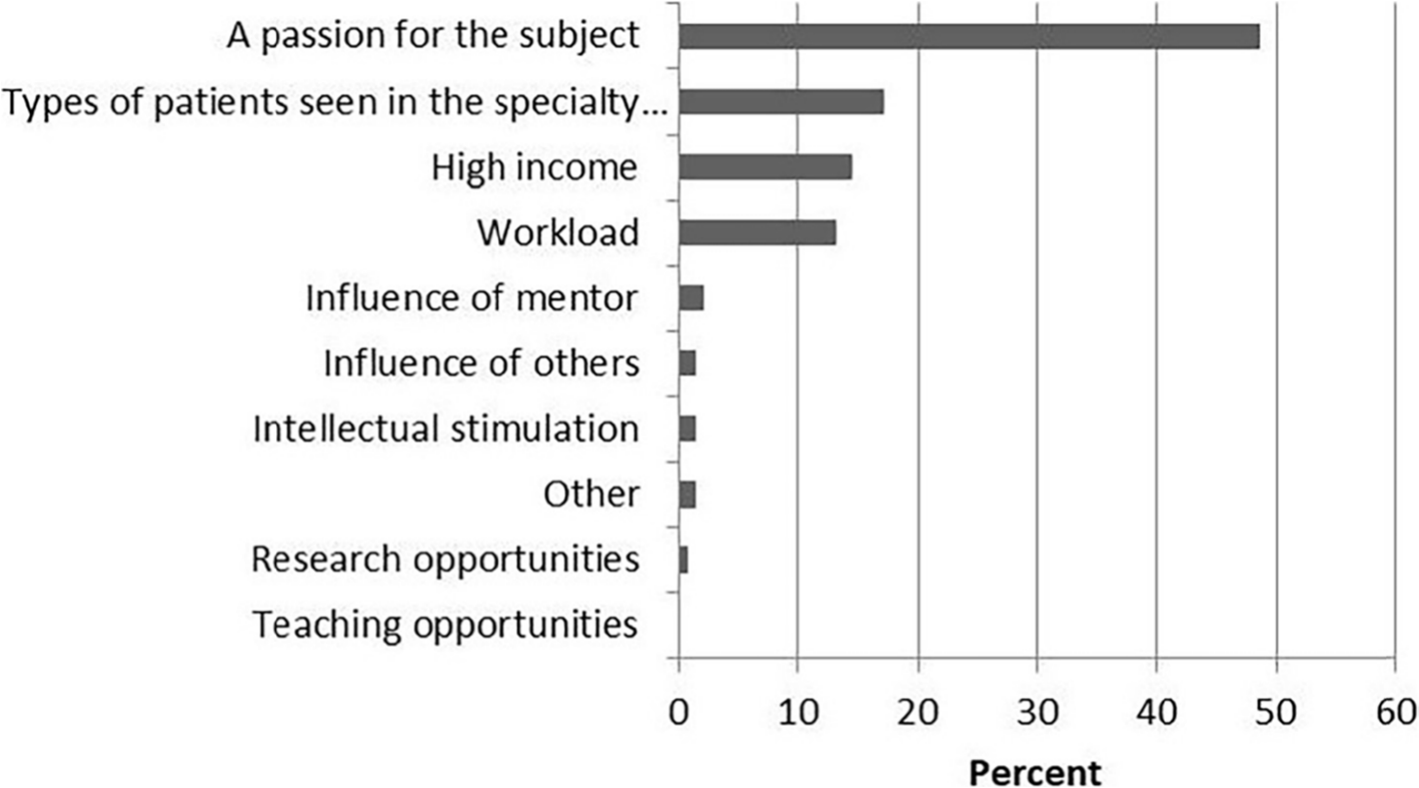
Response to the question: “what was the single most important factor influencing your specialty choice of graduate degree”

When asked to choose main factors that influenced specialty choice of graduate program, the greatest percentage (73.2%) of total respondents chose “a passion for the subject” (Fig. 5). “Types of patients seen in the specialty of service” (48.2%), “high income” (42.1%), “workload” (40.2%), and “influence of mentor” (33.1%) were also ranked as main reasons by respondents. Moreover, when asked to choose the single most important factor influencing their specialty choice of graduate program, similar identifiers were obtained. Again, nearly half of the respondents (48.7%) stated “a passion for the subject” was the single most important factor, as showed in Fig. 6. After this was “Types of patients seen in the specialty of service” (17.1%), “high income” (14.5%) and workload (13.2%).

### Long-term future plans

When asked about the long-term future plan (5 years later), most respondents indicated that they intended to be specialist dentists (52.3%). 45.4% planned to be general dentist, and most of respondents wanted to be occupied in dental education (65.1%) and research work (69.4%). For the work unit with long-term plans, major public hospital (40.0%) and university hospital (23.8%) were their popular choice of work unit with long-term future plan. After these, 16.9% said they would work in private dental offices, and 13.1% said they would register and practice in more than one primary health care institutions as full-time or part-time staff and may also run a private dental office.

### Gender difference in specialty preferences

**Fig. 7:**
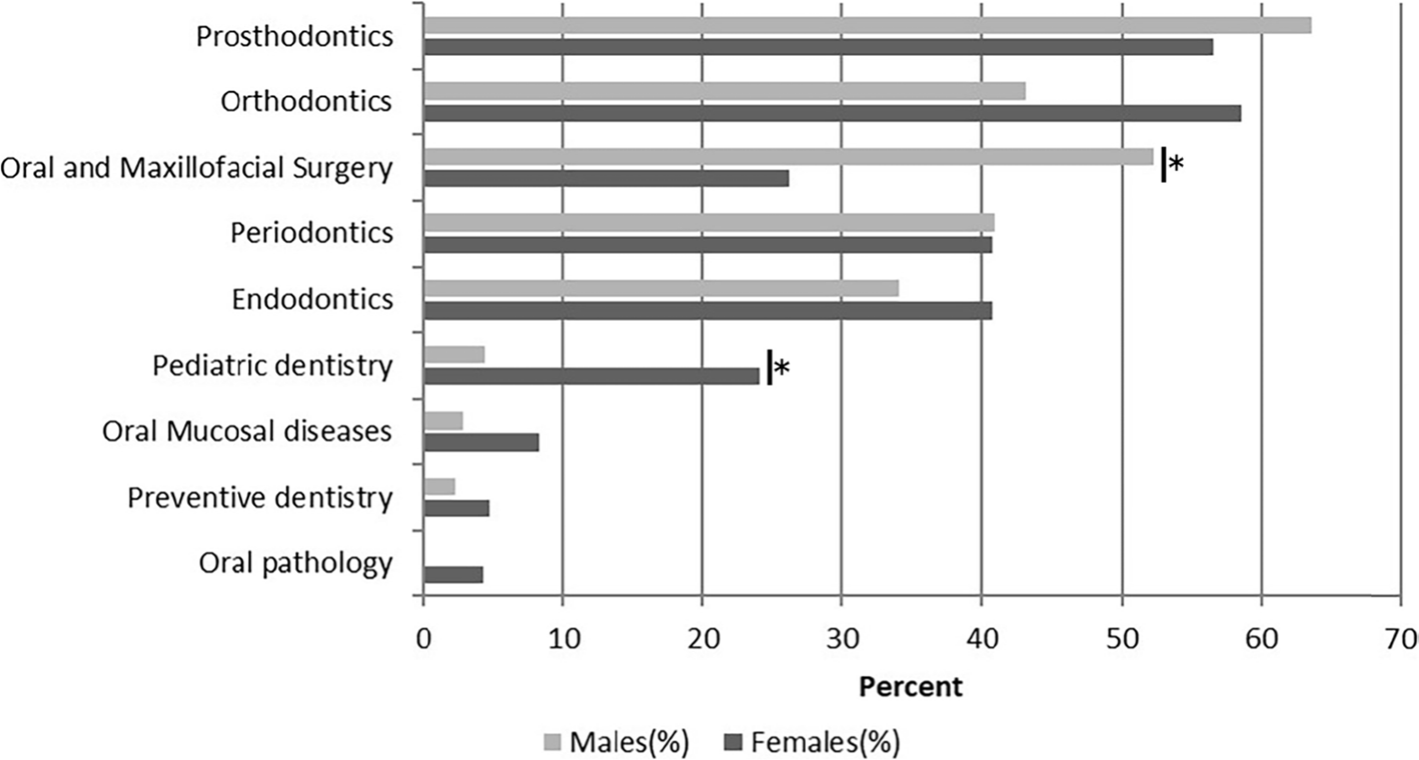
Response to the question: “what were main discipline choices of graduate program. Respondents could select all answers that applied. *p < 0.05. There were significant differences between men and women in the choice of oral and maxillofacial surgery and paediatric dentistry

**Fig. 8:**
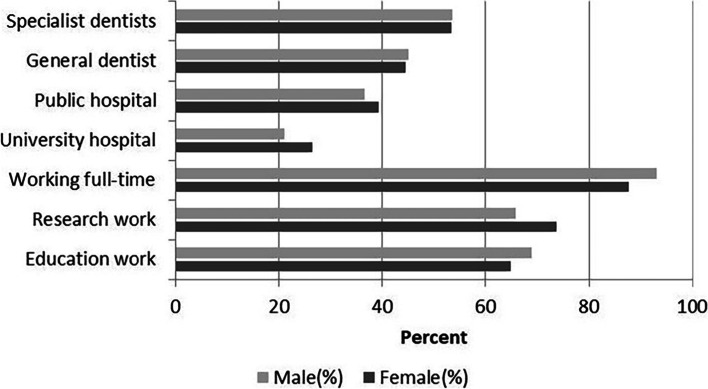
The long-term future plan of female and male

The number of male and female students of final year dental school students from 2016 to 2020 was shown in Table 2. In 2016, there were 60 (66.7%) females and 30 (33.3%) males. And in 2017, females were 41 (71.9%) and males were 16 (28.1%). From the data of the past 5 years, there were fewer males and more females in dentistry. The levels of interest for discipline choices for graduate program by female and male respondents were shown in Fig. 7. Female respondents mainly preferred orthodontics (58.6%), prosthodontics (56.6%), and periodontics (40.7%). In addition, male respondents mainly preferred prosthodontics (63.6%), oral and maxillofacial surgery (52.3%), and orthodontics (43.2%). Gender difference in specialty choice for graduate program among respondents was indicated via Chi-square analysis. Oral and maxillofacial surgery was significantly chosen by male than female (χ2 = 10.493, *P* < 0.05). Paediatric dentistry significantly chosen by male than female (χ2 = 8.23, P < 0.05). The long-term future plan of female and female were showed in Fig. 8. There was no difference between male and female in the long-term future plans. The percentage of females intending to become specialist dentists were 53.2% and that of male 53.5%. 87.7% of female intended to work full-time and 93.0% of male intended to work full-time.

**Table 2:** The number of male and female students of final year dental students from 2016 to 2020

Survey Year	Male, number (%)	Female, number (%)
2016	30 (33.3)	60 (66.7)
2017	16 (28.1)	41 (71.9)
2018	23 (38.3)	37 (61.7)
2019	18 (28.6)	45 (71.4)
2020	24 (38.1)	39 (61.9)
Total	111 (33.3)	222 (66.7)

## Discussion

The survey provided important findings about future plans and the influencing factors, as well as the influence of gender on future choice for the final year students at stomatology school, CMU. This study covered students in five graduating classes from 2016 to 2020, which expanded the number of subjects and made the research results more representative. Our findings suggested that the vast majority of respondents planned to pursue a graduate degree immediately after graduated. Moreover, the main reasons influencing their plans were “eligible for better jobs”. There were gender differences in choice of specialty among dental students. Those who choose paediatric dentistry were almost all females. Female students mainly preferred orthodontics, prosthodontics, and periodontics, while male, mainly preferred oral and maxillofacial surgery, prosthodontics, and orthodontics. To study the workforce trends and future career plans of medical students would help dental educators improve the educational model and policy makers to formulate appropriate policies to meet the country’s future dental health needs and promote the dental profession [21]. This study could raise some useful influence and feedback effect on current health and education policy, and on the career development of practicing dentists or dental students.

This study primarily focused on the survey of final year dental undergraduates at the China medical University, School of Stomatology. The stomatology school of CMU was located in North of China. The dental education at CMU had a history more than 40 years, and the stomatology school and hospital established about 30 years ago. The Chinese Hospital Specialist Reputation Ranking, recognized as the most authoritative ranking in China by far, was based on the evaluation of the specialist reputation and research capability of the major hospitals in China, and published annually. According to the CHSRRs in recent 7 years, stomatology hospital of China medical university ranked 9 or 10 [22] (http://www.fudanmed.com/institute/news222.aspx). The final year dental students have completed the courses of dentistry, and were taking part in the one-year clinical training under the guidance of teachers in a dental clinic. The survey’s results of final year dental undergraduates at the School of Stomatology in CMU reflected the current situation of dental future plans and the influencing factors of dental students in China to some extent.

Selection of a career plan by dental students was a complex and individualized decision. Our study showed that pursue a graduate degree has become the most popular short-term future plan for dental students after graduation, with almost all students choosing to go on to graduate school. The proportion of five-year dental students in China who want to continue to graduate school was much higher than that of other countries. In Britain, only 21.6% of dental students intended to pursue a dental specialty [23] and half of dental students decided not to specialize in USA [24]. There might be two main reasons influencing students’ decision. On the one hand, the implementation of educational policy has led dental students to change their future plans and choices. In order to improve the quality of clinical services on a large scale and effectively alleviate the conflicts between doctors and patients, the Chinese government in 2013 implemented the Standardized Residency Training (SRT) program [25]. SRT in China was a three-year training period after 5 years of undergraduate medical education [26]. As an actively promoted programme, SRT has become the second popular short-term future plan for dental students after graduation. And there were 49.1% of respondents who chose to enter SRT. To some extent, it showed the SRT programme has been accepted by dental and medical students and most of these students are willing to attend to the programme in China. In addition to the regular SRT, the graduate medical and dental education model attempted to combine the clinical master’s degree education with SRT [27]. The clinical master’s degree, which could simultaneously obtain the master’s degree and SRT, was increasingly favoured by undergraduates.

On the other hand, dental students tended to get a better educational background by attending graduate school in order to continually upskill themselves and get a better job. Obtaining a specialist dentist’ license could offer a substantial payoff, with independent dental specialists earning nearly twice as much as general dentists in the USA [28]. Nashleanas et al. [9] thought the earnings gap between general dentists and specialists may tempt students to forgo their current income in favour of specialists. Employers in China’s first-tier cities and/or higher-level hospitals, as well as/or able to raise better salaries, had a growing demand for employees’ educational background and competence. In this study, 52.3% respondents indicated that they intended to be specialist dentists in the long-term; and major public hospital (40.0%) and university hospital (23.8%) were their popular choice of work unit in long-term future plan. The results reflected their expectations for better job opportunities and self-improvement, and might shed some light on why they want to pursue graduate degrees. In addition, there was a noteworthy phenomenon about the time when determining to pursue a graduate degree. Most students (35.9%) determined to pursue a graduate degree before they went to university, which was similar to an American study [24], in which most students (21.0%) determined to specialize before dental school. And more than half of the students decided to pursue a graduate degree before they enter their sophomore year. These meant that the choice to pursue a graduate degree was well thought out and future plans were not made on the spur of the moment.

Orthodontics and prosthodontics were the most popular specialties, while oral pathology was almost never chosen. For those students who wanted to go on to graduate schools, orthodontics and prosthodontics were their favourite specialty, which was similar to a previous study [8]. And a passion for the subject was the first influential factor for those who chose these two specialties. Besides, high income and types of patients seen in the specialty of service were the next most influential factors. This observation was similar to values reported by Nobel et at [29]., wherein passion for specialty’s content and challenges emerged as the most important factor and types of patients seen in the specialty of service was also an important factor. However, 56% students chose orthodontics for high income, which differed from a previous Saudi Arabian study [30], in which none of the students opted for orthodontics because of high income and the vast majority chose orthodontics because of their job satisfaction. Oral pathology was the least popular specialty for dental students. Only 6 out of the 202 students (3%) showed that they were interested in oral pathology, which was similar to a British study [8], in which 3.4% chose oral pathology as first or second choice of specialty. Moreover, in one American study [24], no one chose oral pathology, oral and maxillofacial radiology, and dental public health, which was also consistent with our study. These might be because oral pathology was more research-oriented and could not improve their clinical skills. For the difference in the choice of specialties, we had some recommendations. For those specialties with fewer choices, teachers could actively guide students to make them interested in these specialties through some methods, such as sharing their own clinical experiences and holding tea parties between undergraduates and graduate students in these specialties. Once students are interested in these specialties, teachers could help them understand and learn these specialties deeply and make them fall in love with these specialties. Besides, more social practice activities such as clinical internships could be carried out so that students could have a deep understanding of each specialty and determine whether they really like a specialty, which could reduce students’ regrets in choosing a specialty. Last, the university could organize a regular social practice activity, once every 6 months, which require non-graduating students to learn about the recruitment and employment of various majors at the spring or autumn college student recruitment fairs, and make a brochure about it. In this way, students could have a deep understanding of the labour market and have an estimate of the employment situation they are about to face.

In the long-term future plan, students were willing to do part-time research jobs. 69.4% of respondents in our study wanted to do research work while working as a dentist and 65.1% considered education work in their long-term plans. This was inconsistent with other report [29] that fewer students purse academia was a universal problem in some countries. The phenomenon may have something to do with Chinese professional title evaluation system for doctors, where you have to do academic research if you want to get a more advanced title. And, we could speculate that students would be willing to participate in scientific research and teaching in their future work without affecting the diagnosis and treatment of patients. Thus, appropriate dedicated time to build research careers was likely to be important in order to avoid conflicts between research and service provision [13].

With the trend of feminization, gender difference is becoming the focus of researchers’ attention. In our study, there were far more female students than male students, with 69.5% female and 30.5% male, which were in line with the common gender distribution in dental education [18]. Between 2016 and 2020, the male-female ratio of final year dental undergraduates at the China medical University, School of Stomatology showed a downward trend, with more and more girls. About the specialty preference, male students’ favourite majors were prosthodontics (63.6%) and oral and maxillofacial surgery (52.3%), while female students preferred orthodontics (58.6%) and prosthodontics (56.6%). And this observation was consistent with a British study [8]. Besides, our study found twice as many men as women chose oral and maxillofacial surgery and there were significant differences between men and women in paediatric dentistry (*p* <0.05). Different with previous results [31], in which the preference for specialties did not differ significantly between male and female students, the result showed that gender had a significant influence on the choice of specialty. With the majority of the current population of dental students being female, dentistry specialties would become very unequal in the future labour market. But it also had one benefit. Although there is a shortage of paediatricians in China now, with the feminization of dentistry and women’s choice of paediatric dentistry, the shortage will be greatly reduced in the future. In long-term future plan, there was no significant difference between men and women in terms of full-time and part-time employment, which were different from some studies. In a British study [32], a half (51.80%) of women intended to work part-time compared to males (30.60%) (*p* < 0.05). And females intended to take more time out of their future careers to focus on child care at the University of Bristol [33]. This may be because in China people were used to having their parents help take care of their children, and they went to work as usual. Besides, with the development of society, women spent less time looking after their children in China, while men spent more time looking after their children [34].

In this study, family income, source of tuition and living expenses had no significant impact on students’ short-term and long-term career choice. Surveys conducted by The American Dental Education Association at U.S. dental schools showed the average graduating student had above $200,000 in student debt in 2016 [10]. The debt might have less impact on dental students in China than in other countries. The tuition fee of undergraduate students in China was not expensive, which was similar to other university majors, could be accepted by most families. Influenced by Confucian traditions in Asia, Chinese students’ tuition fees were mostly provided by their families. In this study, 90 % of students’ tuition and living expenses were entirely borne by their parents and family.

This survey was administered to final (five) year students from only one institution for 4 years, which may limit generalizability. Follow-up study needs to base on a greater range to examine whether the findings have wider generalizability. Moreover, only some students who plan to take postgraduate courses passed the exam immediately after graduation. Some of students who *won’t* get the chance to enter the postgraduate programs continued to take this program after failing the exam and take another exam next year, and others changed their plans to go to residency or private practice. Therefore, asking students about short plans immediately after graduation may not accurately reflect what these students actually do after graduation. Further studies of tracking these new dentists for several years after graduation could lead to a better understanding of the career planning of students in Chinese dental schools.

## Conclusion

In this study, we aimed to investigate the short-term and long-term career plans, the influencing factors of future plans, and whether there was a relationship between gender and the future plans for the final year students at stomatology school, China medical university from 2016 to 2020. We found the most students pursued a graduate degree after graduation, with orthodontics and prosthetics becoming the two most popular majors. In the long-term plan, students were willing to become specialist dentists and work part-time in research and teaching. The percentage of males choosing oral and maxillofacial surgery were twice as high as that of females, and almost all the students choosing paediatric dentistry was females. Several limitations existed in this survey.

## Supplementary Information

**Additional file 1.**

## Data Availability

All data generated or analysed during this study are included in this published article and its supplementary information files.
